# Reactions in Lymph Nodes Draining X-Irradiated and Carcinogen Treated Tissues

**DOI:** 10.1038/bjc.1956.35

**Published:** 1956-06

**Authors:** A. H. E. Marshall

## Abstract

**Images:**


### REACTIONS IN LYMPH NODES DRAINING X-IRRADIATED AND CARCINOGEN TREATED TISSUES

A. H. E. MARSHALL*

* Freedom Research Fellow.

*From the Bernhard Baron Institute of Pathology, The London Hospital, E.*1

Received for publication March 23, 1956

A NUMBER of authors have described changes in lymphoid tissue after irradiation or exposure to carcinogenic agents. These reactions appear to be of two distinct types. Firstly, formation of so-called "haemo-lymph nodes" may occur. This change has been described, among others, by Clarkson, Mayneord and Parsons (1938) following whole body X-irradiation in mice, and by Lasnitski and Woodhouse (1944) following treatment with a number of carcinogens. It is probable that the increase of iron pigment observed by Hoch-Ligeti (1941) in the nodes of mice treated with 1, 2, 5, 6-dibenzanthracene was a late stage of this effect.

The second change consists of the formation of plasma cells throughout the lymphoid tissue. This reaction has been reported following whole-body exposure to ionizing radiation in man (Liebow, Warren and De Coursey 1949), in dogs (Wohlwill and Jetter, 1953), in monkeys (Schlumberger and Vazquez, 1954), in mice following irradiation and also after treatment with a water soluble carcinogen (Parsons, 1943), and in the mesenteric nodes of mice following intraperitoneal injection of carcinogenic hydrocarbons (Green, 1954). In view of the known connection between plasma cell proliferation and antibody formation (Fagraeus, 1948), the last change was interpreted by Green to represent an immune reaction against altered protein formed by the carcinogen and to be intimately connected with the subsequent process of tumour formation.

In this paper the reaction of the regional lymph nodes to local treatment with carcinogens and X-rays was studied in mice and guinea-pigs at varying periods from the time of application of the agents.

### METHODS

### *Carcinogens*

9, 10-Dimethyl-1, 2-benzanthracene and 20-methylcholanthrene dissolved in acetone were employed.

Fifty Swiss albino mice were given weekly paintings with a 0·15 per cent solution of 9, 10-D.M.B.A. with 0·2 per cent methylcholanthrene on the right foreleg, thoracic and axillary regions. These animals were killed at intervals of from 14 days to 14 weeks from the first painting. The right and left axillary groups of lymph nodes were removed, weighed, and retained for histological study. Superficial ulceration of the skin of the treated area occurred in 8 of 50 animals, and tumour formation was present in 7 of the 10 animals surviving at 14 weeks.

A group of 8 animals was also given a single painting with 0·4 per cent 9, 10-D.M.B.A. and sacrificed 14 days later. Severe skin damage occurred in these animals.

*X-rays*

*Group* 1.—Five female guinea-pigs were given a total of 9,000r of X-rays (50 KV., 3 Ma., H.V.L. 0·33 mm. Al., 4 cm. field, 4 cm. distance, 3,500r per min.) in 5 separate doses of 1,500r at 2–3 week intervals, on the dorsum of one foot and were killed 10 days after the last exposure.

*Group* 2.—Five female guinea-pigs were also given a single exposure on one foot of 2,900r and a second exposure of 2,800r after a 5-week interval. They were killed from 4 to 10 days after the second exposure. Following the second exposure ulceration occurred in 4 of 5 animals ; in this group such animals were killed within 48 hours of the appearance of ulcers.

In mice treated with chemical carcinogens the axillary lymph nodes on both the treated and control sides were removed, weighed and fixed in formol alcohol. In irradiated guinea-pigs, the popliteal and femoral lymph glands on the irradiated and normal sides were similarly removed. Portions of the carcinogen-treated and irradiated skin were also preserved for histological study. Paraffin sections from this material were stained by haematoxylin and eosin, Unna-Pappenheim's method and Perl's method for iron.

### RESULTS

All mice treated with 20-methylcholanthrene and 9–10 dimethyl benzanthracene, and killed from 14 days to 14 weeks, showed a marked increase in weight of the axillary lymph nodes on the treated side (Table I). This increase was greatest in the 7 animals bearing tumours after 14 weeks' treatment.

TABLE I—*Total Weights of Axillary Lymph-nodes from Control and Treated Sides in Mice Painted for Varying Periods with Carcinogen.*

| Carcinogen. | Duration (days). | Treated weight (mg.). | Control weight (mg.). |
|---|---|---|---|
| D.M.B.A. | 14 | 430 | 270 |
| D.M.B.A. | 21 | 320 | 160 |
| D.M.B.A. | 35 | 205 | 100 |
| 20-methylcholanthrene | 42 | 170 | 70 |
| 20-methylcholanthrene | 98 | 160 | 60 |
| 20-methylcholanthrene (tumour bearing) | 98 | 450 | 180 |

### *Histological Changes in Lymph Nodes Draining Carcinogen Treated Areas*

These reactions were identical whether 20-methylcholanthrene or 9, 10-dimethyl-benzanthracene were used. They may be described under two headings.

(1) *Reactions in cortical and medullary pulp of nodes*

These reactions developed within 14 days of the start of treatment and showed only a moderately greater degree in animals killed after long periods of treatment. The main proliferative reaction was in the medullary pulp of the node and showed in its earlier stages a diffuse proliferation of cells with pale oval nuclei and inconspicuous non-basophilic cytoplasm (Fig. 1). These cells resemble the " primitive reticular cells " described by many authors as being the common stem cell observed in lymphoid and myeloid tissues. In material studied after longer periods of treatment, fewer cells of the primitive reticular type were present and a considerable degree of differentiation to small and medium lymphocytes appeared to have occurred (Fig. 2). The resulting proliferation produced a considerable widening of the medulla of the node with some compression and narrowing of the medullary cords near the hilum. In non-tumour bearing animals treated with carcinogens from 14 days to 7 weeks there was no evidence of plasma cell proliferation in the medullary cords or medullary pulp of the node, and the few plasma cells of mature type present in the medullary cords did not exceed that observed in the control node from the opposite side. In 3 mice (non-tumour bearing) treated for 14 weeks with methylcholanthrene, however, a moderate plasma cell increase was present in the medullary cords of the treated node.

The formation of germinal centres, which forms a prominent part of the lymph node reaction to antigens, was not conspicuous in the treated animals ; hyperplastic centres were present in both control and treated nodes of some animals, and were absent in treated nodes in others.

In 7 tumour-bearing animals treated with methylcholanthrene a moderate to heavy plasma cell proliferation was present in the medullary cords of the treated nodes (Fig. 3) in addition to the lymphocytic medullary proliferation previously described.

(2) *Reactions in sinuses of treated nodes*

These were similar to those previously described by Lasnitski and Woodhouse (1944). A moderate proliferation of sinus histiocytes occurred, and in some animals red cells were present, either free or intracellularly. Free iron could also be demonstrated in the cytoplasm of the histiocytes in many treated animals. This change was not confined to the nodes draining the treated area but was also present in control glands from the opposite axilla.

### *Histological Changes in Lymph Nodes Draining X-irradiated Tissue*

*Group* 1

In 5 guinea-pigs given a total of 9,000r of X-rays in 5 doses of 1,500r, the popliteal and femoral nodes draining the radiated foot showed only slight histological changes. Slight proliferation of sinus lining histiocytes was present in all examples, in one animal considerable phagocytosis of red cells was present. A moderate hyperplasia of the lymphoid tissue of the medulla occurred in all animals. This apparently produced only cells of the lymphocyte series, and resembled the proliferation seen in carcinogen treated animals. A small proliferation of mature plasma cells was present in the medullary cords in the popliteal node of one animal.

*Group* 2

In the 5 guinea-pigs given a single exposure of 2,900r, followed after 5 weeks by a second exposure of 2,800r, more marked histological changes were present in the draining lymph nodes. A similar sinus proliferation to that seen in Group 1

was present, and polymorphonuclear leucocytes, red corpuscles and a few eosinophils were present. Lymphoid proliferation similar to Group 1 was also present in the medulla of the node (Fig. 4). A marked plasma cell proliferation occurred in the medulllary cords of the lymph nodes of 4 animals (Fig. 5). Plasma cells of immature type were present in these proliferations, some showing mitotic figures.

### DISCUSSION

Of the various reactions described in the treated lymph nodes, that of deposition of iron pigment or phagocytosis of red cells appears to correspond with that previously described by a number of authors. This reaction would appear to be a consequence of local or general capillary damage by X-irradiation or chemical carcinogens with subsequent leakage of red cells into the tissue spaces.

The hyperplasia of stem cells with subsequent lymphocytic differentiation which occurred in the medulla of nodes draining both X-irradiated and carcinogen-treated tissues does not appear to have been previously observed. The reaction resembles that seen in human nodes following a variety of infective and inflammatory stimuli, but in view of present ignorance of the functions of lymphocytes in such processes, no assessment of its significance is possible.

The generalized formation of plasma cells in lymphoid or myeloid tissue has been described following exposure to irradiation or general absorption of an injected carcinogen. It is known that considerable bacteraemia may follow whole-body irradiation, and in view of the frequently observed production of plasma cells by antigen injection it is possible that the plasma cell proliferation in irradiated animals is a secondary consequence of bacteraemia. A similar mechanism may account for the production of plasma cell proliferation in animals following parenteral injection of carcinogens. In these experiments, in which both small and heavy doses of carcinogen were applied to the skin, there was no satisfactory evidence of plasma cell proliferation in the draining lymph node; such proliferation was present however in animals bearing tumours in the treated areas.

The formation of plasma cells as a local response to ionizing radiation has been reported as a sequela of radiotherapy of tumours in man, where it forms part of the so-called " stromal reaction ". Some authors (Koller, 1947) have considered this reaction to be of considerable importance in determining the response of the tumours to radiotherapy. The significance of this reaction is uncertain as some degree of infection of the tumour might produce a similar result. In the experiments reported here, plasma cell proliferation was observed in some instances in draining lymph nodes following local exposure to too heavy doses of X-rays and in which

---

### EXPLANATION OF PLATE.

FIG. 1.—Reaction in medulla of regional lymph node after 21 days treatment with methylcholanthrene. Diffuse proliferation of primitive reticular cells, and more mature lymphocytic elements. H. & E. × 700.

FIG. 2.—Late stage of reaction in medulla of methylcholanthrene treated node. The proliferation of mature lymphocytes is compressing the remaining sinus tissue of the node. H. & E. × 120.

FIG. 3.—Plasma cell reaction in medullary cords of regional node in a tumour-bearing animal. No metastasis was present in the node. H. & E. × 500.

FIG. 4.—Proliferation of small and medium lymphocytes in medulla of lymph node draining X-irradiated area of guinea-pig foot. H. & E. × 120.

FIG. 5.—Plasma cell proliferation in medulla of guinea-pig lymph node draining X-irradiated foot. H. & E. × 450.

1

2

3

4

5

local irradiation damage was most severe, but was not seen when a larger total dose was given in 5 fractionated amounts. These results do not show any constant plasma cell response to local irradiation of normal tissue but suggest that such a response may occur when severe necrosis of tissue takes place either as a consequence of antigenic substances derived from damaged tissue or from bacterial contamination of the radiated area.

### SUMMARY

In 58 albino mice given weekly paintings on one side of the chest and axilla with 9, 10-dimethyl-1, 2-benzanthracene or 20-methylcholanthrene, an enlargement of the regional nodes, characterized by proliferation of lymphocytic cells in the medulla, was constantly produced. There was no evidence of plasma cell proliferation in response to carcinogen treatment alone, although some such proliferation occurred in animals bearing tumours.

In 10 guinea-pigs treated with multiple doses of X-rays to one foot, a similar lymphocytic hyperplasia was observed; plasma cell proliferation also occurred in the regional nodes in animals in which severe local tissue necrosis had occurred.

### REFERENCES

Clarkson, J. R., Mayneord, W. V. and Parsons, L. D.—(1938) *J. Path. Bact.*, **46**, 221.
Fagraeus, A.—(1948) *Acta med. scand.*, Suppl. 204.
Green, H. K.—(1954) *Brit. med. J.*, **2**, 1374.
Hoch-Ligeti, C.—(1941) *Cancer Res.*, **1**, 484.
Koller, P. C.—(1947) *Brit. J. Radiol.* Suppl. 1, p. 84.
Lasnitski, A. and Woodhouse, D. L.—(1944) *J. Anat. Lond.*, **78**, 121.
Liebow, A. A., Warren, S. and De Coursey, E.—(1949) *Amer. J. Path.*, **25**, 853.
Parsons, L. D.—(1943) *J. Path. Bact.*, **55**, 397.
Schlumberger, H. G. and Vazquez, J. J.—(1954) *Amer. J. Path.*, **30**, 1013.
Wohlwill, F. J. and Jetter, W. W.—(1953) *Ibid.*, **29**, 721.


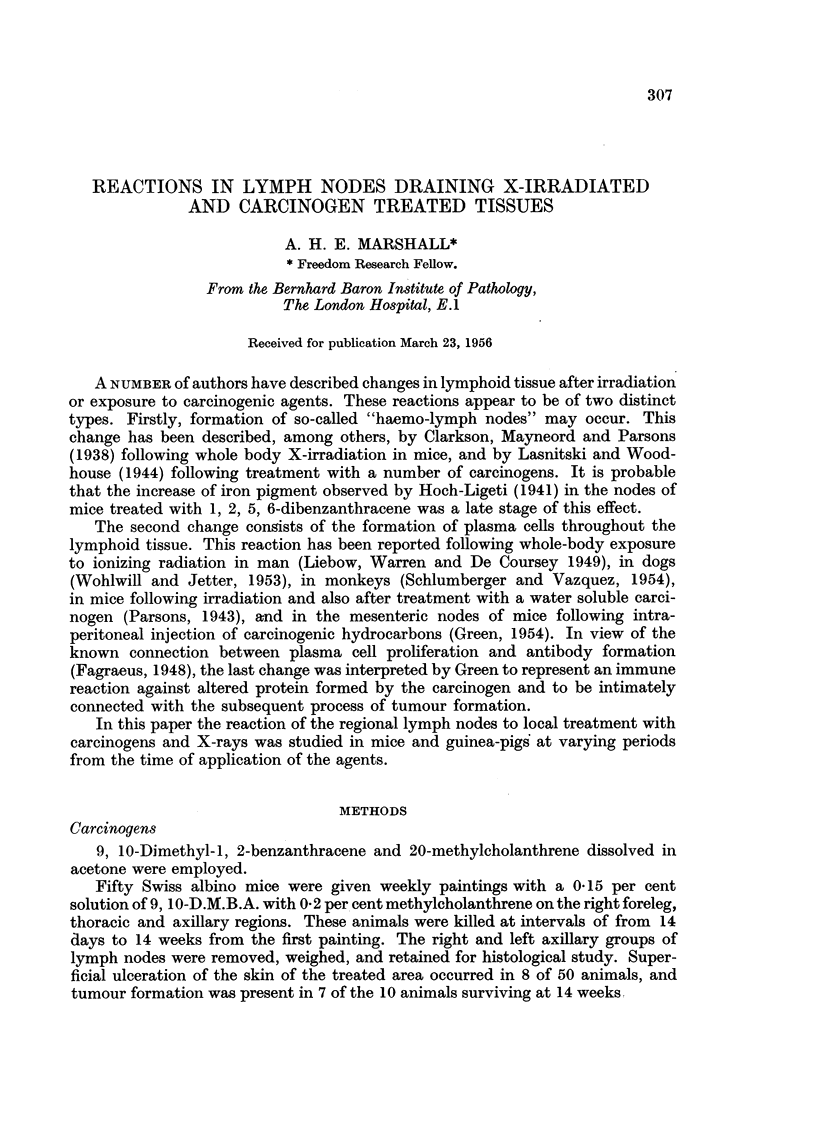

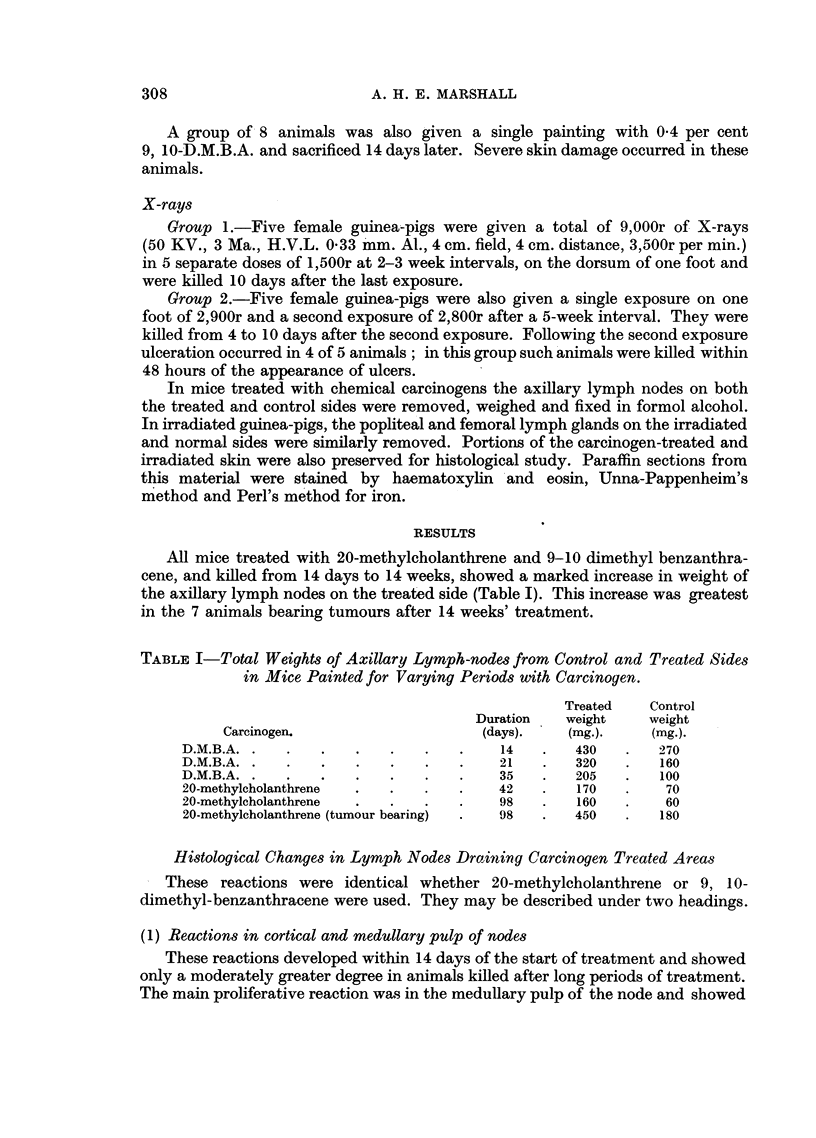

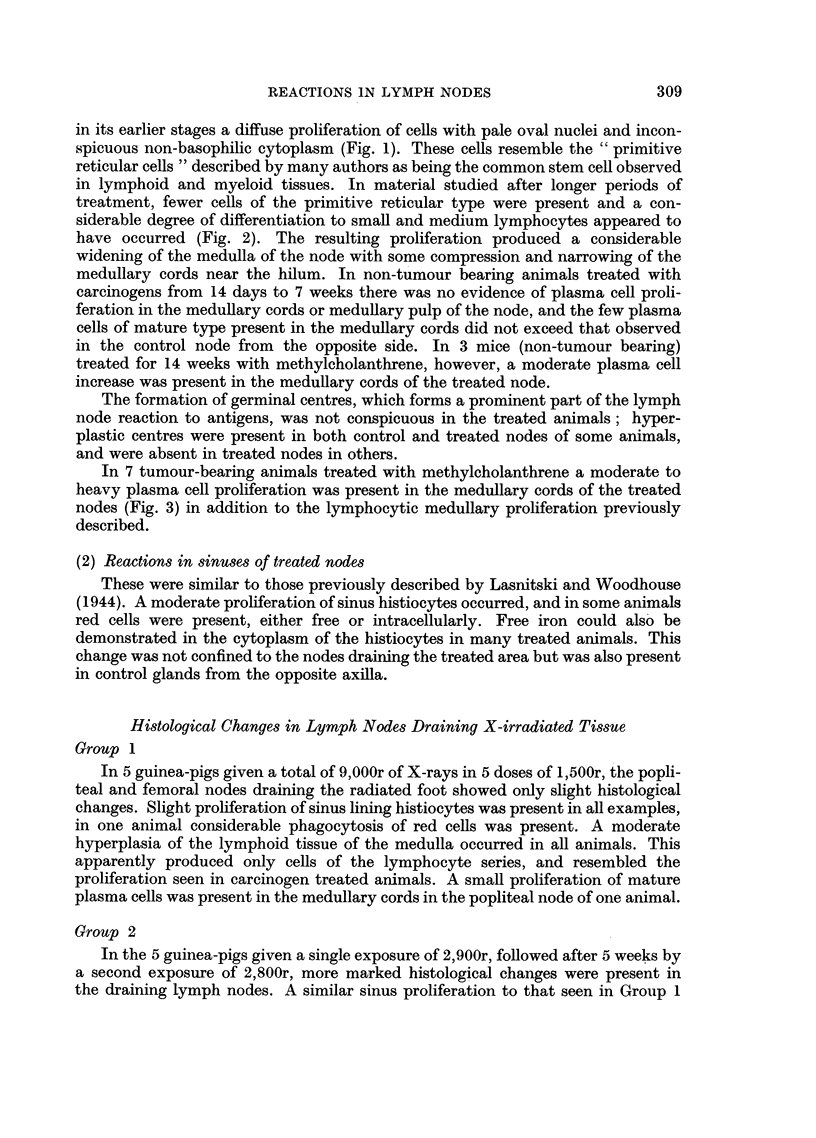

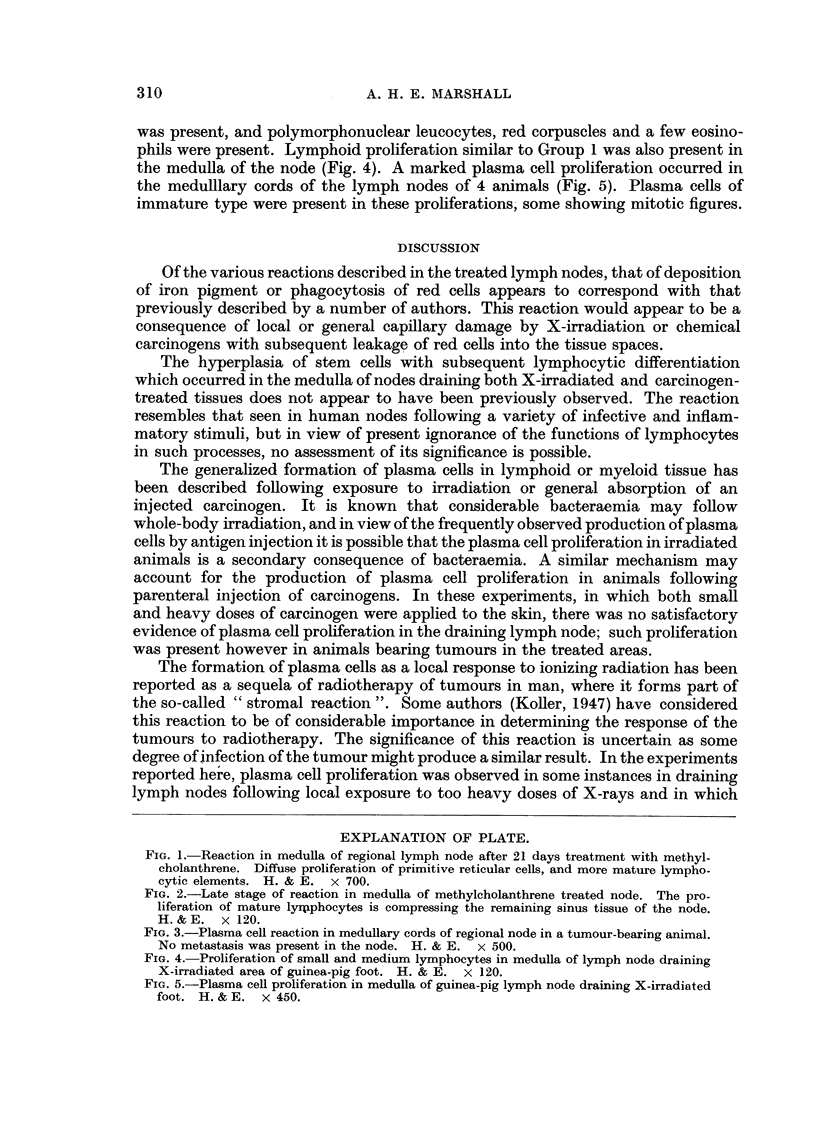

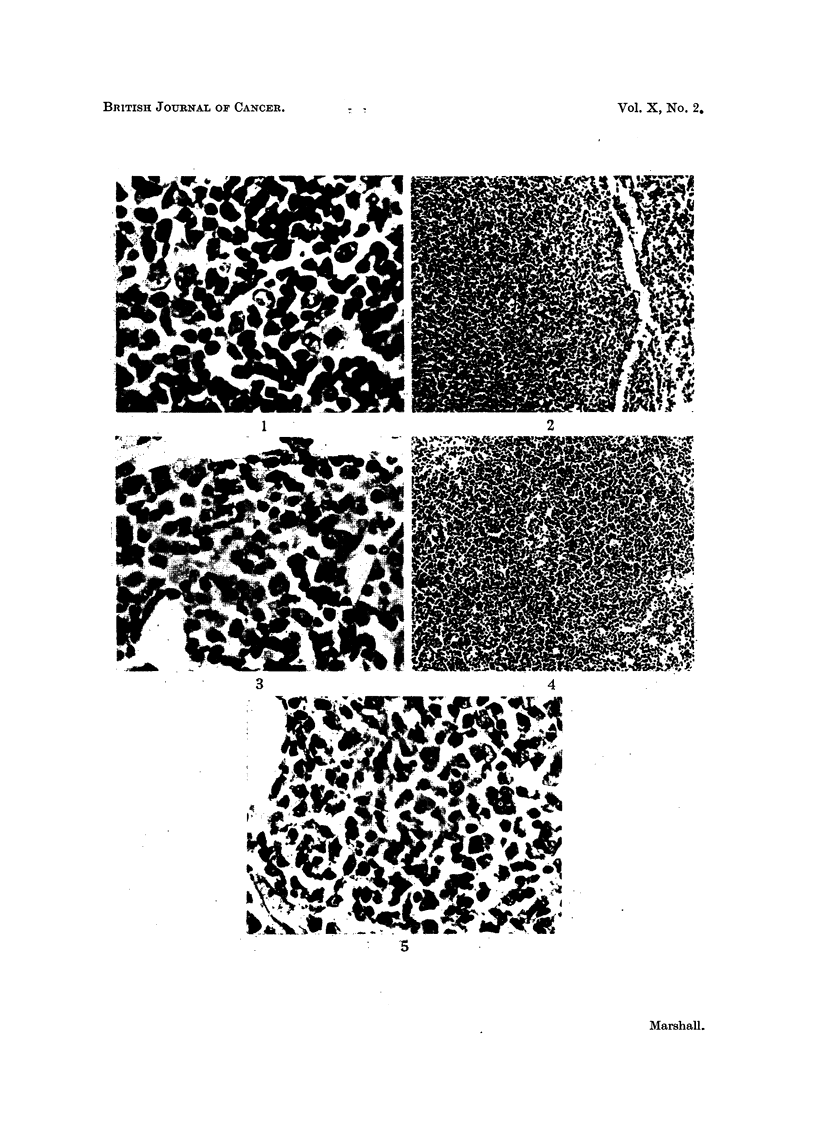

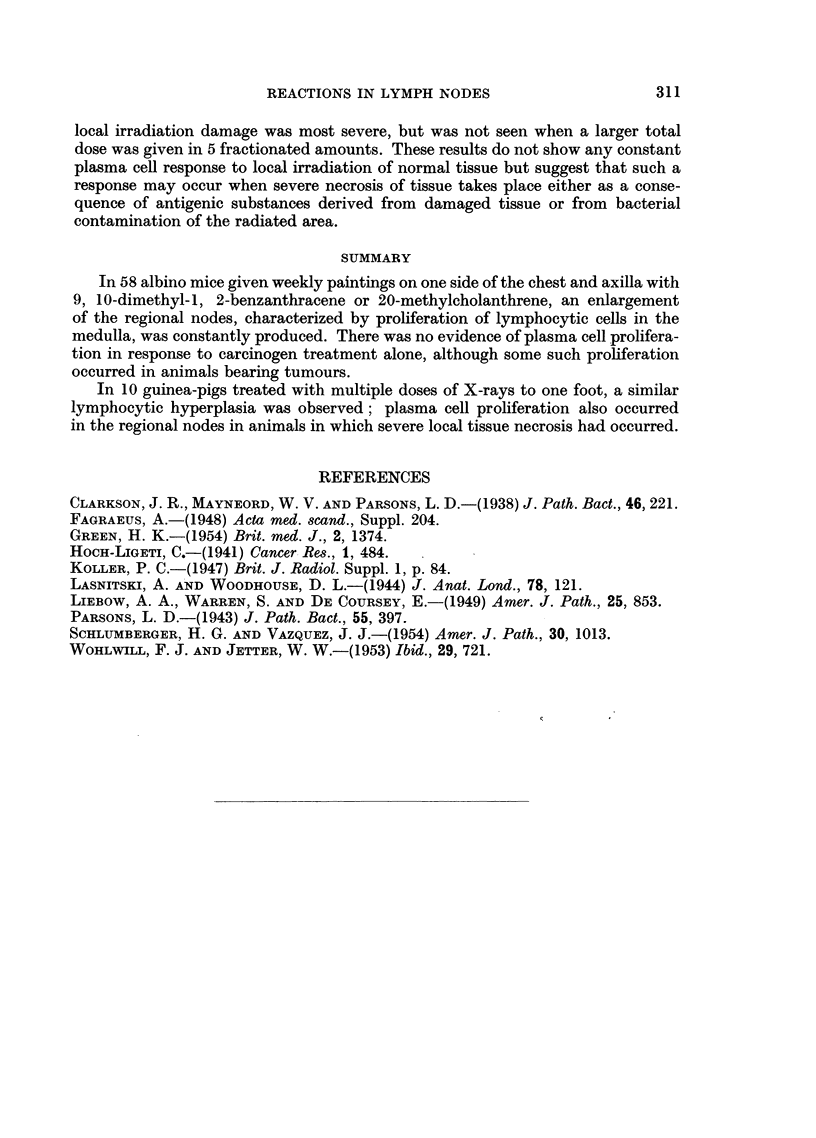


## References

[OCR_00287] GREEN H. N. (1954). An immunological concept of cancer: a preliminary report.. Br Med J.

[OCR_00294] LIEBOW A. A., WARREN S., DeCOURSEY E. (1949). Pathology of atomic bomb casualties.. Am J Pathol.

[OCR_00292] Lasnitzki A., Woodhouse D. L. (1944). The effect of 1:2:5:6-dibenzanthracene on the lymph-nodes of the rat.. J Anat.

[OCR_00297] SCHLUMBERGER H. G., VAZQUEZ J. J. (1954). Pathology of total body irradiation in the monkey.. Am J Pathol.

[OCR_00298] WOHLWILL F. J., JETTER W. W. (1953). The occurrence of plasma cells after ionizing irradiation in dogs.. Am J Pathol.

